# A large-scale survey of genetic copy number variations among Han Chinese residing in Taiwan

**DOI:** 10.1186/1471-2156-9-92

**Published:** 2008-12-24

**Authors:** Chien-Hsing Lin, Ling-Hui Li, Sheng-Feng Ho, Tzu-Po Chuang, Jer-Yuarn Wu, Yuan-Tsong Chen, Cathy SJ Fann

**Affiliations:** 1 Department of Life Sciences and Institute of Genome Sciences, National Yang-Ming University, Taipei, Taiwan; 2 Institute of Biomedical Sciences, Academia Sinica, Taipei, Taiwan

## Abstract

**Background:**

Copy number variations (CNVs) have recently been recognized as important structural variations in the human genome. CNVs can affect gene expression and thus may contribute to phenotypic differences. The copy number inferring tool (CNIT) is an effective hidden Markov model-based algorithm for estimating allele-specific copy number and predicting chromosomal alterations from single nucleotide polymorphism microarrays. The CNIT algorithm, which was constructed using data from 270 HapMap multi-ethnic individuals, was applied to identify CNVs from 300 unrelated Han Chinese individuals in Taiwan.

**Results:**

Using stringent selection criteria, 230 regions with variable copy numbers were identified in the Han Chinese population; 133 (57.83%) had been reported previously, 64 displayed greater than 1% CNV allele frequency. The average size of the CNV regions was 322 kb (ranging from 1.48 kb to 5.68 Mb) and covered a total of 2.47% of the human genome. A total of 196 of the CNV regions were simple deletions and 27 were simple amplifications. There were 449 genes and 5 microRNAs within these CNV regions; some of these genes are known to be associated with diseases.

**Conclusion:**

The identified CNVs are characteristic of the Han Chinese population and should be considered when genetic studies are conducted. The CNV distribution in the human genome is still poorly characterized, and there is much diversity among different ethnic populations.

## Background

The human genome contains many DNA sequence variations, including single nucleotide polymorphisms (SNPs), short nucleotide insertions or deletions, tandem repeat sequences, and transposable elements [1]. Recent human genome studies have revealed that copy number variations (CNVs) are more common than previously thought. Some CNVs are associated with gene expression levels and thus may contribute to phenotypic differences [2-7]. Each CNV is a DNA segment > 1 kb that shows variation within the population in terms of a deletion and/or an amplification. Although SNPs are regarded as the main source of phenotypic differences among humans, CNVs also have a large impact on differential gene expression [2].

Microarray-based approaches, so called "molecular karyotyping", have been used to detect subtle chromosomal structure variations on the genome-wide scale [8]. SNP microarrays are high-resolution tools for genotyping that can be used to simultaneously detect copy number (CN) alterations and loss-of-heterozygosity [9,10]. The copy number inferring tool (CNIT), an algorithm recently developed for use with Affymetrix GeneChips, efficiently predicts regions with subtle CN changes and is based on a hidden Markov model (HMM) [11]. CNIT had higher accuracy and lower variation in CN estimation than other programs, including the copy number analysis tool [9] and the copy number analysis with regression and tree approach [12]. In this study, 100 K GeneChip intensity data from 270 HapMap multi-ethnic individuals were used to determine the parameters of the CNIT algorithm. Intensity data from 300 normal unrelated individuals were then used to predict CNV regions using CNIT.

Stringent selection criteria were used to classify true and false CN-altered predictions: CN-altered regions found in at least two individuals were classed as CNV regions. A total of 230 copy number-variable regions (CNVRs) were identified in the sample population, 64 of which (27.83%) had a CNV allele frequency ≥ 1%. Of these 230 CNVRs, 133 (57.83%) had previously been reported in the genomic variant database . The CNVRs ranged from 1.48 kb to 5.68 Mb (mean = 322 kb), and contained 449 genes and 5 microRNAs (miRNAs). Sixty-three CNVRs (27.39%) were associated with segmental duplications (SDs), which are known to induce non-allelic homologous recombination and produce structural variations.

## Results

### Detection of regions with copy number alterations

**Figure 1:**
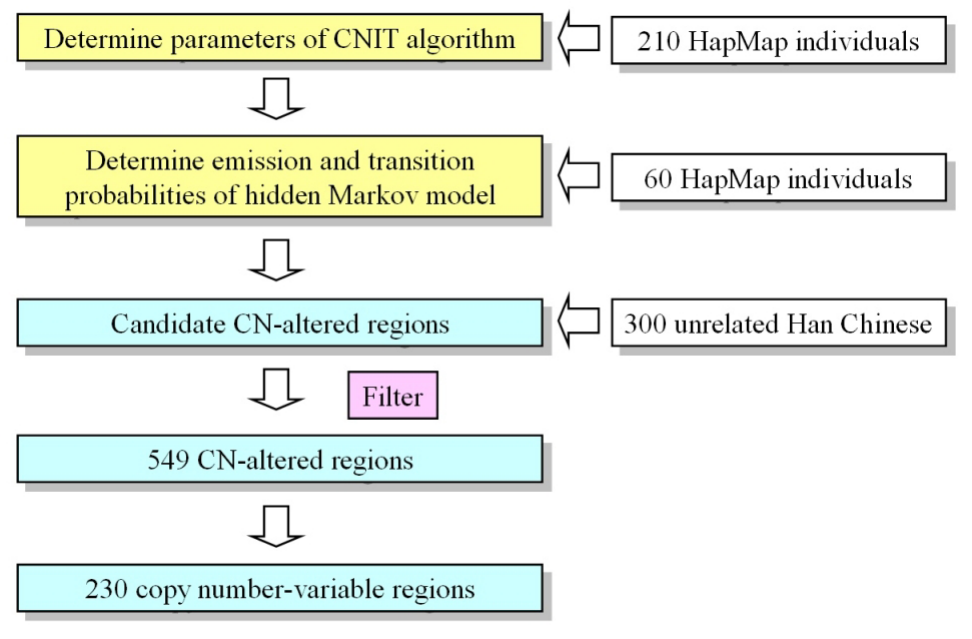
**A flowchart of this study**. The parameters of the copy number inferring tool (CNIT) algorithm for single nucleotide polymorphism (SNP) copy number (CN) estimations were determined using a reference group that consisted of 210 unrelated, multi-ethnic HapMap individuals. The emission and transition probabilities of the hidden Markov model were determined using data from 40 HapMap individuals, and were verified using data from 20 additional HapMap individuals. After construction of the CNIT algorithm, data from 300 unrelated Han Chinese individuals in Taiwan were used to predict candidate CN-altered regions. A total of 549 reliable CN-altered regions were identified; 230 regions showed copy number variations (CNVs) in at least two individuals.

**Figure 2:**
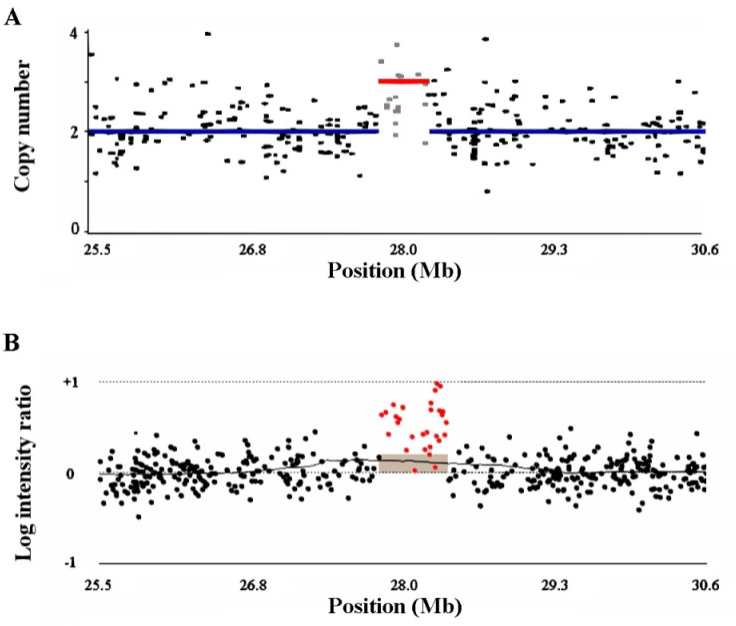
**GeneChip and comparative genomic hybridization (CGH) array data from a Han Chinese individual revealing a single copy-number (CN)-altered segment on chromosome 21**. (A) Affymetrix 100 K GeneChip analysis using the copy number inferring tool (CNIT). The size of the amplified segment (red) was 373 kb. There were 17 SNPs within this region (mean CN = 2.70). (B) Agilent 244 K CGH array analysis. The size of the amplified segment (shaded box) was 512 kb based on a threshold z-score of 2.5.

In this study, 646 samples and 5 complicated processes were used to detect genetic regions with CN alterations. A flowchart of the study design is presented in Figure 1. First, the Affymetrix 100 K GeneChip intensity data from HapMap 270 multi-ethnic individuals were used to construct the CNIT algorithm. The intensity data from 376 unrelated Han Chinese individuals in Taiwan were used for CNV region identification. Although the SNP call rate was one of the quality control indexes for the GeneChip experiments, 76 of the 376 Han Chinese individuals (20%) having larger numbers of CN-altered regions were excluded from the following analyses. Using the CNIT algorithm, a total of 13,729 CN-altered events were predicted in the 300 individuals. Certain factors intrinsic to GeneChips are thought to affect probe intensity, so additional criteria were used to eliminate false-positive results. For example, sequence variations within restriction-enzyme cutting sites or SNP probe sequences can directly alter SNP probe intensity and reduce the accuracy of CN estimation. Therefore, quantitative PCR (qPCR) experiments were used to validate more than 200 CNIT-predicted events from the first selection round and the results were analyzed. The following criteria were then used to eliminate false-positive CN-altered findings: (1) the number of consecutive SNPs was required to be ≥ 6 and < 15 with *P *≤ 0.05; or (2) the number of consecutive SNPs was required to be ≥ 15. Using these additional criteria, the false-positive rate (FPR) of the validated data was reduced from 0.3 to 0.005; however, 34.15% of true CN-altered events were lost. After filter selection, a total of 4,288 CN-altered events in 549 genomic regions were retained. As an additional measure of cross-platform validation, results from Agilent 244 K comparative genomic hybridization (CGH) arrays from two selected individuals were compared to the results from the CNIT predictions and results were consistent between the two arrays. An example of one extra copy of a genomic segment of chromosome 21 is shown in Figure 2.

### Regions of copy number variants among Han Chinese population

**Figure 3:**
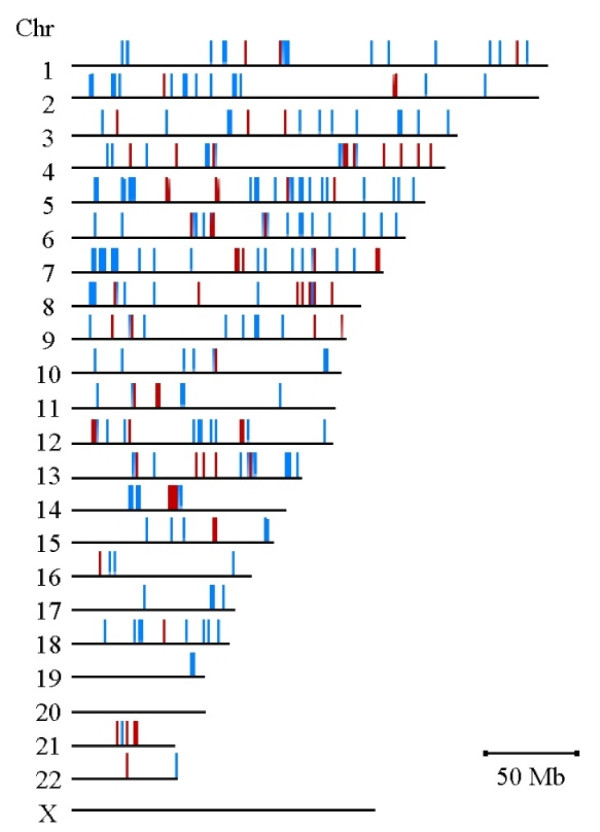
**The copy number variation (CNV) map of a Han Chinese population in Taiwan**. There were 230 CNV regions (blue) identified from 300 unrelated individuals. Of these regions, 64 had greater than 1% CNV allele frequency and were classed as copy-number polymorphic regions (red).

Among the 300 individuals, 76 and 224 DNA samples were extracted from peripheral blood and cell lines, respectively. It is known that subtle chromosomal abnormalities can be induced during cell culture. Although all these cell lines were freshly cultured, some chromosomal abnormalities might still have been generated during culture. To exclude this possibility, only those CN-altered regions that were identified in more than two individuals were classed as CNVRs. Of the 549 CN-altered segments, 230 were classified as CNVRs in the Han Chinese population (Additional File 1). Among these 230 CNVRs, 133 (57.83%) had been reported previously and 97 (42.17%) were unique to this study. Ten of these novel CNVRs (10%) were validated using qPCR. Of the 230 CNVRs, 196 (85.22%) had simple deletion alleles, 27 (11.74%) had simple amplification alleles, and 7 (3.04%) were comprised of both deletion and amplification alleles. These CNVRs ranged from 1.48 kb to 5.68 Mb (mean = 322 kb) and covered a total of 2.47% of the human genome. As shown in Figure 3, these CNVRs are widely dispersed throughout the genome, with the exception of chromosomes 20 and X. Moreover, 64 of the 230 CNVRs had a CNV allele frequency greater than 1%. Of the CNVs in this study, 27% were associated with SDs, similar to previous results (24%) [5].

The individuals in this study shared the same ancestry as HapMap individuals from Beijing, so the CNV regions identified among Han Chinese in Taiwan were compared with those identified among Beijing residents [5]. Sixty CNV regions from the 45 unrelated HapMap Beijing individuals were detected using the 100 K GeneChip and CNIT algorithm, and 12 (20%) of these were also observed in the samples of this study.

### Genes within copy number variation regions

There were 449 genes located within 81 of the 230 CNVRs, and most of these genes (56.35%) had simple amplifications (Additional File 2). The 449 genes identified are involved in many biological functions, including ion transport, metabolism, and cell-surface functions. Some of the identified genes are involved in potassium and/or sodium transport, such as the *KCNT1*, *KCNJ8*, *SLC2A6 *and *SLC16A3 *genes, and some are known to be involved in metabolism, such as the *AMY *genes that catalyze the first step in digestion of dietary starch and glycogen [13]. The copy numbers of the *AMY1 *gene are positively correlated with salivary amylase protein levels, which might result in differences in the digestion of starchy foods among individuals [14].

MicroRNAs (miRNAs) have recently been shown to be involved in the regulation of development and physiology in animals and plants [15]. miRNAs can specifically recognize target mRNAs with complementary sequences and cause translational repression or site-specific cleavage of the target. Five miRNAs in four CNVRs were identified in this study (Table 1) and all had previously been reported to have variable CN [5,7]. Six target genes of these miRNAs had been predicted using the MIRANDA software , but none had been validated. Some CN-variable genes have been shown to be correlated with diseases (Table 2), including the egl nine homolog 1 (*EGLN1*) and dopamine beta-hydroxylase (*DBH*) genes. The *EGLN1 *gene is located on chromosome 1q42.1. Deficiency of this gene causes familial erythrocytosis type 3 (ECYT3) (MIM# 609820), which is characterized by increased serum hemoglobin and hematocrit levels, but normal serum erythropoietin levels. Defects in the *DBH *gene cause DBH deficiency (MIM# 223360), which is characterized by marked deficits in autonomic and cardiovascular function.

**Table 1:** MicroRNAs in the copy number-variable regions (CNVRs)

**CNVR ID**	**Chr**	**Position (bp)**	**Variant Type**	**# of observations^*a*^**	**miRNA^*b*^**	**Target Gene (*P *value)^*c*^**
102	7	3488259–5891682	Loss and Gain	4	*hsa-mir-589*	
127	8	14713230–14862195	Loss	2	*hsa-mir-383*	SNCG (9.71 × 10^-6^)
143	9	72363501–72631779	Loss	2	*hsa-mir-204*	NELF (1 × 10^-6^) PLXDC2 (7.1 × 10^-6^) XYLT1 (1.5 × 10^-7^)
212	17	72497067–78181864	Gain	5	*hsa-mir-338*	
					*hsa-mir-657*	BDKRB2 (6.7 × 10^-7^) HOXB13 (7.1 × 10^-6^)

^*a *^Total number of gains and losses observed for a CNVR.

^*b *^MicroRNA overlapping a CNVR based on microRNAs of the UCSC March 2006 assembly.

^*c *^Target gene of microRNA based on MIRANDA software analysis using a 1 × 10^-5 ^cut-off [19].

**Table 2:** Genes in the copy number-variable regions (CNVRs) are associated with disease and disease susceptibility

**CNVR ID**	**Chr**	**Position (bp)**	**Variant Type**	**# of observations^*b*^**	**Gene symbol^*c*^**	**Disease^*d*^**
1^*a*^	1	17313441–19641958	Gain	4	*ALDH4A1*	Hyperprolinemia, type II
					*MRT4*	Mental retardation
					*PAX7*	Rhabdomyosarcoma, alveolar
13^*a*^	1	229469402–229804452	Loss	11	*EGLN1*	Erythrocytosis, familial, 3
					*GNPAT*	Chondrodysplasia punctata, rhizomelic, type 2
31^*a*^	3	15281603–15545420	Gain	8	*COLQ*	Endplate acetylcholinesterase deficiency
38	3	130171189–130741211	Loss	2	*GP9*	Bernard-Soulier syndrome, type C
					*RHO*	Retinitis pigmentosa 4
40^*a*^	3	166493869–167384443	Loss	4	*BCHE*	Apnea, postanesthetic
80	5	147335938–147460716	Loss	2	*SPINK5*	Atopy
111	7	80115926–80209630	Loss	10	*CD36*	Platelet glycoprotein IV deficiency
120^*a*^	7	142035481–142155613	Loss	2	*PRSS1*	Trypsinogen deficiency
121^*a*^	7	154254541–155708987	Gain	6	*SHH*	Coloboma, ocular
124^*a*^	8	13016678–13270959	Loss and Gain	4	*DLC1*	Colorectal cancer
147	9	103072622–103206674	Loss	3	*BAAT*	Hypercholanemia, familial
149^*a*^	9	134892783–137859477	Gain	8	*ADAMTS13*	Thrombotic thrombocytopenic purpura, familial
					*CEL*	Maturity-onset diabetes of the young, type VIII
					*COL5A1*	Ehlers-Danlos syndrome, type I
					*DBH*	Dopamine beta-hydroxylase deficiency
					*SURF1*	Leigh syndrome, due to COX deficiency
152^*a*^	10	49832039–50706976	Gain	5	*CHAT*	Myasthenic syndrome, congenital, associated with episodic apnea
					*ERCC6*	Cerebrooculofacioskeletal syndrome 1
156^*a*^	10	125651321–126729328	Loss	2	*OAT*	Gyrate atrophy of choroid and retina with ornithinemia
163^*a*^	12	2449181–3410784	Gain	6	*CACNA1C*	Timothy syndrome
164	12	4794562–5039110	Gain	3	*KCNA1*	Episodic ataxia/myokymia syndrome
167^*a*^	12	21603812–21931308	Loss	8	*ABCC9*	Cardiomyopathy, dilated, 1O
					*GYS2*	Glycogen storage disease, type 0
					*KCNJ8*	Prinzmetal angina
					*LDHB*	Lactate dehydrogenase-B deficiency
176^*a*^	13	22656237–23918863	Gain	2	*SACS*	Spastic ataxia, Charlevoix-Saguenay type
					*SGCG*	Muscular dystrophy, limb-girdle, type 2C
206	16	11215600–11869359	Loss	4	*LITAF*	Charcot-Marie-Tooth disease, type 1C
212^*a*^	17	72497067–78181864	Gain	5	*ACTG1*	Deafness, autosomal dominant 20/26
					*ASPSCR1*	Alveolar soft-part sarcoma
					*FSCN2*	Retinitis pigmentosa-30
					*GAA*	Glycogen storage disease II
					*SGSH*	Sanfilippo syndrome, type A
					*SOCS3*	Dermatitis, atopic, 4
215^*a*^	18	26241506–27419753	Loss	5	*DSC2*	Arrhythmogenic right ventricular dysplasia, familial, 11
					*DSG1*	Keratosis palmoplantaris striata I
					*DSG2*	Arrhythmogenic right ventricular dysplasia, familial, 10
					*DSG4*	Hypotrichosis, localized, autosomal recessive
217^*a*^	18	51204895–51803567	Loss	4	*TCF4*	Pitt-Hopkins syndrome
230^*a*^	22	47187524–49023156	Gain	4	*ALG12*	Congenital disorder of glycosylation, type Ig
					*MLC1*	Megalencephalic leukoencephalopathy with subcortical cysts

^*a *^CNVRs reported in the database of genomic variants.

^*b *^Total number of gains and losses observed for a CNVR.

^*c *^Gene overlapping a CNVR based on ReqSeq of the UCSC March 2006 assembly.

^*d *^Disease or disease susceptibility associated with the gene, according to the OMIM Morbid Map.

## Discussion

In this report, a newly developed algorithm, CNIT, was used to identify CNVRs in a normal Han Chinese population. A key assumption of the CNIT algorithm construction is that the CN value of each SNP in the reference group should be two; however, this is not always realistic. Some SNPs with CN aberrations were excluded before constructing the CNIT algorithm due to the availability of CNV data from HapMap individuals. When these CNVs in the reference group were excluded, the identification rate of known CNVs in the test group increased from 23.30% to 25.46%. Owing to the stringent criteria in this study (that is, the parameters of the HMM method and the selection criteria), the false-negative rate (FNR) might be high, resulting in an underestimation of the CNV allele frequency. Nevertheless, these stringent criteria largely reduced the FPR and yielded reliable CNV results. Higher resolution microarrays or other technological approaches are needed to further address these structural variations in detail.

Most of the CNVRs (85.22%) identified in this study were simple deletions; only a few were simple amplifications (11.74%). The divergence might be due to the emission probability (EP) of the HMM method in the CNIT algorithm. The intensity distribution in a cell line with three copies of the X chromosomes was examined using 100 K GeneChip data and the CNIT algorithm. Intensity differences between samples containing gene amplifications (CN = 3) and those containing normal CNs (CN = 2) were less distinct than the difference between samples containing hemizygous deletions (CN = 1) and normal CNs (CN estimations for one, two and three copies of X chromosomes were 1.38 ± 0.42, 2.08 ± 0.47 and 3.03 ± 1.49, respectively). To eliminate false-positive findings, a stringent EP was used for amplifications (the EP for amplifications was the reverse of the EP for deletions), and as a result, some CNVs with amplifications might have been lost in this CNV survey.

In this large-scale survey of CNVs in a Han Chinese population in Taiwan, 97 CNVRs (42.17%) were unique; other CNVRs have previously been reported in Asian populations [5]. In the current genomic variant database, most CNVs are rare (allele frequency < 3%). Fewer than 200 CNVRs had an allele frequency of > 5% and these CNVRs were detected using different microarray platforms such as Affymetrix 500 K GeneChips and CGH arrays. The probe distribution of the 100 K GeneChip data was quite different from the 500 K GeneChip or CGH array data. Therefore, it was difficult to precisely compare the results across different platforms. The reliability of the CNVRs found in this study was supported by stringent selection criteria, agreement with previous independent studies, qPCR analysis and CGH experiments.

Some CN-variable genes identified in this study are known to be correlated with diseases, but individuals in the current study were healthy. Disease models and environmental factors are critical to disease etiology, perhaps explaining why the individuals with gene CN changes were healthy. The well-developed CNIT algorithm for 100 K GeneChip data can be used in future studies to identify subtle chromosome abnormalities .

## Conclusion

CNVs have recently been recognized as an important structural variation in the human genome. In this study, 42.17% of the identified CNVRs were novel, indicating that the CNV loci of the human genome are still not fully understood and that there is much diversity among different ethnic populations. Most CNV allele frequencies were low, and only two CNVRs had a frequency greater than 10%. This observation is consistent with results reported previously by Jakobsson *et al *[4]. The CNVs reported in this study are characteristic of Han Chinese populations and should be considered when genetic studies are conducted.

## Methods

### SNP microarray intensity data

Data from 376 unrelated individuals were randomly collected from the Taiwanese Cell and Genome Bank [16]; the raw microarray intensity data have been described in previous studies [11,17]. Individual genotyping was performed by the National Genotyping Center (Academia Sinica, Taipei, Taiwan) using the Affymetrix 100 K GeneChip Human Mapping set (Affymetrix, Santa Clara, CA, USA) according to the manufacturer's instructions. The SNP call rate of these samples examined in this study was 98.04 ± 0.8%.

In addition, the intensity data from 100 K GeneChip microarrays of 270 individuals were downloaded from the HapMap project website ; the CNV data for these individuals were collected previously [5]. These individuals included 30 Caucasian trios, 30 African trios and 90 unrelated Asian individuals. The HapMap reference group, which included 210 multi-ethnic unrelated individuals, was used to determine the parameters of CNIT for SNP CN estimation. Forty offspring were used as a training group for the HMM method, and 20 additional offspring were used as a test group to evaluate the FPR and FNR.

### Copy Number Inferring Tool

The CNIT algorithm showed good performance in CN estimation of SNPs and DNA segments [11], and well-characterized HapMap samples were used to determine the parameters for CNIT. In both single-point and multipoint CN estimations, results obtained from CNIT showed greater accuracy and reduced FPR and FNR compared with that obtained from other programs. When the smoothing procedure was not used, CNIT could detect small CN-altered regions that were missed with other programs. SNPs located in the reported CNV regions of the corresponding individuals were removed, such that the CN for most of the autosomal SNPs from the reference, training and test groups was two. The 100 K GeneChip consists of two chips, each with > 50,000 SNPs (58,960 for the *Xba*I chip and 57,244 for the *Hind*III chip). The reference group was used to process probe selection, estimate the coefficient of preferential amplification/hybridization, and construct a CN distribution for each SNP [11]. After probe selection, 94,587 SNPs (mean intermarker distance = 30.9 kb) were used to represent CN dosage. Finally, EP and transition probabilities of the HMM method were determined using the training and test groups, and were used to identify true CN-altered regions and eliminate false-positive CN inference by considering contiguous SNPs [11].

### CNV validations

Primer Express Software version 3.0 (Applied Biosystems, Foster City, CA, USA) was used to design PCR primers for the selected target CNVs. Quantitative PCR experiments were performed using the ABI PRISM 7900 Sequence Detector (Applied Biosystems). PCR reactions were performed using the Power SYBR-Green PCR reagent kit (Applied Biosystems) and each reaction mix (25 μl total) contained 2.5 ng genomic DNA for each CNV. qPCR comprised initial denaturation at 94°C for 3 minutes, 40 cycles of denaturation at 94°C for 15 seconds, and a combination of annealing and extension at 60°C for 60 seconds. The fluorescence signal was detected in real-time during the qPCR procedure. The primer pair for the long interspersed nuclear elements 1 (LINE1) sequence was used for normalization [18]. The mean estimated CN was calculated from triplicate PCR reactions for each individual.

The Agilent 244 K CGH array (Agilent Technologies, Palo Alto, CA, USA) contains 236,000 coding and non-coding sequences at a resolution of 6.4 kb. Amplified DNA (2 μg) was used for Cy5/Cy3 labeling according to the manufacturer's protocol. After array hybridization and scanning, CN-altered regions were identified using CGH analytics 3.4 software (Agilent Technologies) with a threshold z-score of 2.5.

### Statistical analysis

After CN estimation for each SNP, CN-altered regions were predicted using the HMM package  based on single-point results. The means of SNP *P *values and CN represent the statistical significance and CN of state-changed segments, respectively. The above data analyses were performed using SAS/STAT version 8 software (SAS Institute, Cary, NC, USA).

## Abbreviations

CGH: comparative genomic hybridization; CN: copy number; CNIT: copy number inferring tool; CNV: copy number variation; CNVR: copy number-variable region; DBH: dopamine beta-hydroxylase; ECYT3: erythrocytosis type 3; EGLN1: egl nine homolog 1; EP: emission probability; FNR: false-negative rate; FPR: false-positive rate; HMM: hidden Markov model; LINE1: long interspersed nuclear elements 1; miRNA: microRNA; SNP: single nucleotide polymorphism; SD: segmental duplication.

## Authors' contributions

CHL carried out this study and drafted the manuscript. LHL and TPC provided experimental assistance, and SFH, JYW and YTC provided analytic and data assistance. CSJF provided analytic support and supervised the project. All authors have read and approved this final manuscript.

## Supplementary Material

Additional file 1 **Copy number-variable regions (CNVRs) and individual copy number variations (CNVs) in this study.** The data contain the list of CNVRs and individual CNVs found in this study. Click here for file

Additional file 2 **Genes in the copy number-variable regions (CNVRs).** The data contain the genes with variable copy number in this study. Click here for file
